# Chronic treatment with paeonol improves endothelial function in mice through inhibition of endoplasmic reticulum stress-mediated oxidative stress

**DOI:** 10.1371/journal.pone.0178365

**Published:** 2017-05-31

**Authors:** Ker Woon Choy, Yeh Siang Lau, Dharmani Murugan, Mohd Rais Mustafa

**Affiliations:** Department of Pharmacology, Faculty of Medicine, University of Malaya, Kuala Lumpur, Malaysia; Max Delbruck Centrum fur Molekulare Medizin Berlin Buch, GERMANY

## Abstract

Endoplasmic reticulum (ER) stress leads to endothelial dysfunction which is commonly associated in the pathogenesis of several cardiovascular diseases. We explored the vascular protective effects of chronic treatment with paeonol (2'-hydroxy-4'-methoxyacetophenone), the major compound from the root bark of *Paeonia suffruticosa* on ER stress-induced endothelial dysfunction in mice. Male C57BL/6J mice were injected intraperitoneally with ER stress inducer, tunicamycin (1 mg/kg/week) for 2 weeks to induce ER stress. The animals were co-administered with or without paeonol (20 mg/kg/oral gavage), reactive oxygen species (ROS) scavenger, tempol (20 mg/kg/day) or ER stress inhibitor, tauroursodeoxycholic acid (TUDCA, 150 mg/kg/day) respectively. Blood pressure and body weight were monitored weekly and at the end of treatment, the aorta was isolated for isometric force measurement. Protein associated with ER stress (GRP78, ATF6 and p-eIF2α) and oxidative stress (NOX2 and nitrotyrosine) were evaluated using Western blotting. Nitric oxide (NO) bioavailability were determined using total nitrate/nitrite assay and western blotting (phosphorylation of eNOS protein). ROS production was assessed by *en face* dihydroethidium staining and lucigenin-enhanced chemiluminescence assay, respectively. Our results revealed that mice treated with tunicamycin showed an increased blood pressure, reduction in body weight and impairment of endothelium-dependent relaxations (EDRs) of aorta, which were ameliorated by co-treatment with either paeonol, TUDCA and tempol. Furthermore, paeonol reduced the ROS level in the mouse aorta and improved NO bioavailability in tunicamycin treated mice. These beneficial effects of paeonol observed were comparable to those produced by TUDCA and tempol, suggesting that the actions of paeonol may involve inhibition of ER stress-mediated oxidative stress pathway. Taken together, the present results suggest that chronic treatment with paeonol preserved endothelial function and normalized blood pressure in mice induced by tunicamycin *in vivo* through the inhibition of ER stress-associated ROS.

## Introduction

The endoplasmic reticulum (ER) is the cellular organelle which is responsible for protein translation, biosynthesis, translocation, folding and post-translational modifications including glycosylation, disulfide bond formation, and chaperone-mediated protein folding processes [1]. When ER homeostasis or function is impaired by biological stress such as ATP deprivation, hypoxia or calcium overload, this will lead to the accumulation of unfolded proteins [2]. Following this, glucose-regulated protein 78 (GRP78) is released, permitting their oligomerization to deal with accumulated unfolded proteins which activates transcriptional and translational pathways known as the unfolded protein response (UPR) [3]. When UPR is activated, 3 distinct UPR branches are initiated namely protein kinase-like ER kinase (PERK) which phosphorylates eukaryotic translation initiation factor 2 alpha (eIF2α), the inositol requiring kinase 1 (IRE1), and the activating transcription factor 6 (ATF6) [4]. Excessive and prolonged UPR will activate pro-apoptotic pathway which contribute to the development of cardiovascular diseases [5]. ER-initiated apoptosis is mediated through IRE1 and CHOP (C/EBP-homologous protein), either by downregulation of BCL-2 (anti-apoptotic protein) or interrupting calcium haemostasis signalling [6]. ER stress-induced ROS and apoptosis was demonstrated in animal model of arteriosclerosis [6], ER stress [7], hypercholesterolemia [8] and diabetes [9]. Therefore, targeting UPR component molecules and reducing ER stress will be promising strategies to treat cardiovascular diseases.

Recent studies demonstrate a synergistic relationship between ER stress and oxidative stress in the pathogenesis of cardiovascular diseases [4, 10]. ER stress pathway involving calcium and Ca^2+^/calmodulin-dependent protein kinase II (CaMKII) has been shown to activate nicotinamide adenine dinucleotide phosphate (NADPH) oxidase, leading to oxidative stress [7, 11]. NADPH, a multi-subunit enzymatic complex, is one of the key generating sources of cellular reactive oxygen species (ROS) such as superoxide anion (O_2_^−^) in the vasculature [12, 13]. Nitric oxide is released by the endothelium and causes vascular relaxation [14]. However, O_2_^−^ acts as a vasoconstrictor and reacts rapidly with nitric oxide (NO), forming peroxynitrite which in turn leads to eNOS uncoupling to produce more O_2_^−^ [15]. ROS-producing enzymes such as NADPH, xanthine oxidase, cyclooxygenase, inactivation of the antioxidant system, and uncoupling of endothelial NO synthase lead to oxidative stress [16]. Excessive production of oxidants causes increased peripheral resistance which have been implicated in the development of hypertension [17]. Oxidative stress-mediated hypertension is associated with inactivation of NO [18]. These processes induce intracellular calcium build up, initiation of inflammatory signalling pathways and increased extracellular matrix deposition, leading to endothelial dysfunction in hypertension [19–21]. Therefore, searching for natural products with antioxidants properties should have beneficial effects in a reduction in blood pressure.

**Fig 1:**
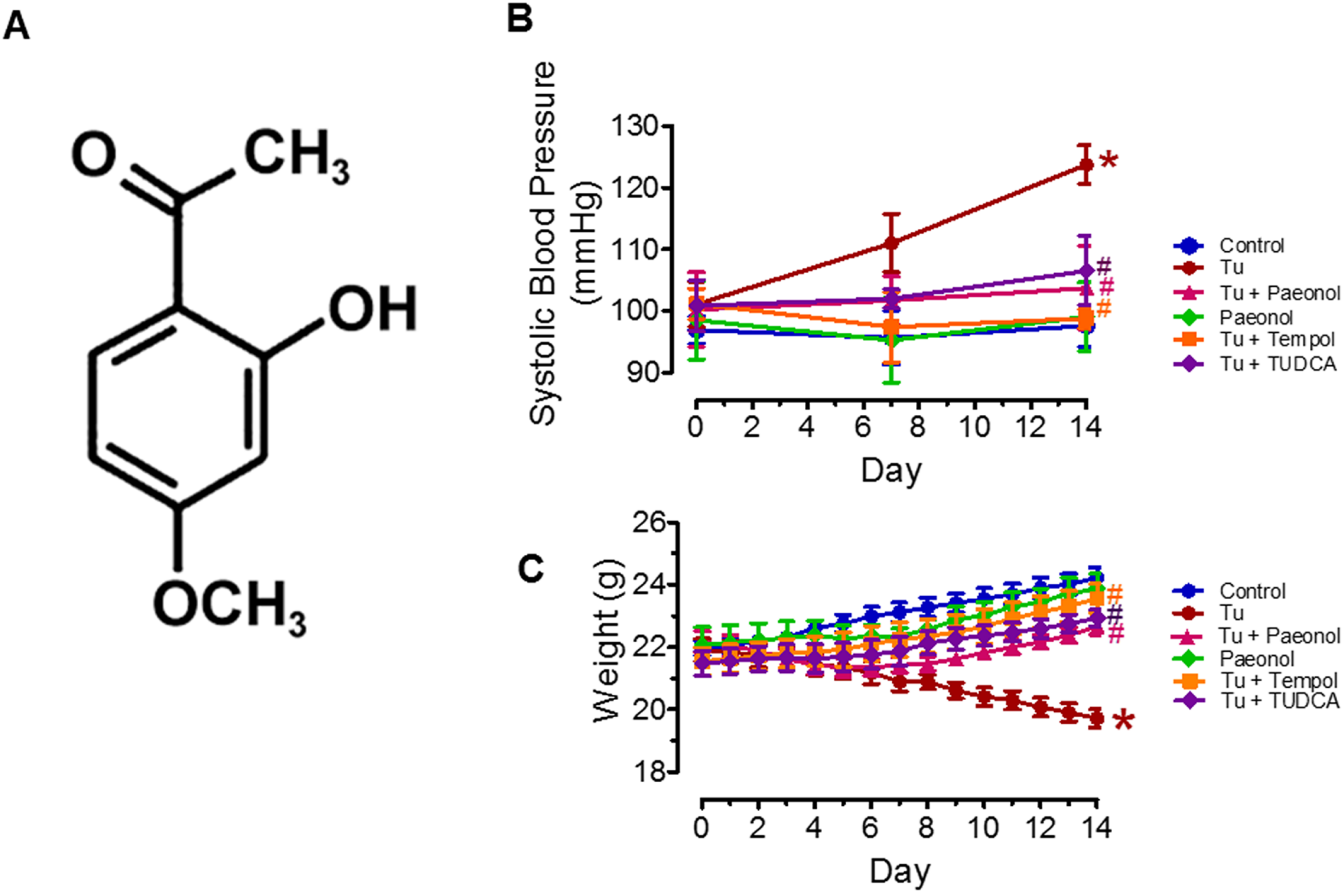
(A) Chemical structure of paeonol. (B) Systolic blood pressure (SBP) and (C) body weight (g) measured in all groups of C57BL/6J mice treated 2 weeks with or without intra-peritoneal injection of tunicamycin (Tu, 1 mg/kg, 2 injections/week) and co-treated with paeonol (20mg/kg/2 weeks/oral gavage), tempol (20 mg/kg/day/oral gavage) and TUDCA (150 mg/kg/day/ip) respectively. Results are means ± SEM of 6–7 separate experiments. *P<0.05 compared with control, # P<0.05 compared with tunicamycin.

Paeonol or 2’-hydroxy-4’-methoxyacetophenone (Fig 1A) is the main phenolic compound of a Chinese herbal medicine which is prepared from the root bark of the plant *Paeonia suffruticosa* Andrew. Paeonol is used in traditional oriental medicines to improve blood circulation, amenorrhea, dysmenorrhea and fever [22, 23]. Paeonol has been previously reported to protect against acetaminophen-induced hepatotoxicity in mice [24], improved Parkinson's disease in mouse model [25] and diabetic encephalopathy in streptozotocin-induced diabetic rats by attenuating oxidative stress [26]. Previously, we reported that paeonol protects against ER stress-induced endothelial dysfunction via inhibition of the upstream pathway involving 5′ adenosine monophosphate-activated protein kinase (AMPK)/peroxisome proliferator-activated receptor δ (PPARδ) signalling in an *in vitro* model [27]. However, the chronic effects of paeonol on ER stress-induced oxidative stress resulting in endothelial dysfunction and increased blood pressure *in vivo* remains obscure. Therefore, the present study seek to investigate the endothelial protective effects of paeonol against ER stress-mediated ROS overproduction and elevation of blood pressure in mice. We hypothesized that chronic treatment of paeonol for 2 weeks protects against ER stress-induced oxidative stress and normalised blood pressure. The results of this investigation may provide new insights for the role of paeonol to mitigate ER stress related cardiovascular diseases such as hypertension, heart failure, ischemic heart diseases, and atherosclerosis.

## Materials and methods

### Materials

Tunicamycin, paeonol (H35803, purity 99%), TUDCA, acetylcholine (ACh) chloride, sodium nitroprusside (SNP), phenylephrine, Tween-20, bis-N-methylacridinium nitrate (lucigenin), diethylthiocarbamic acid (DETCA), diphenylene iodonium (DPI), β-NADPH and Tris-base were purchased from Sigma-Aldrich (St. Louis, MO, USA). Bovine serum albumin (BSA) was purchased from Santa Cruz (Dallas, Texas, USA). Kreb’s salts were purchased from BDH Limited and BDH Laboratory Supplies (Poole, UK). Tempol was purchased from Tocris (Bristol, UK). All the chemicals were dissolved in double distilled water.

### Animals and experimental protocol

All experiments were performed with approval from Institutional Care and Use Committee (IACUC) of University of Malaya (Ethics reference no: 2016-170531/PHAR/R/MRM). Male C57BL/6J mice (8-weeks-old) weighed (mean±SD) 22±14 grams were purchased from Monash University (Sunway Campus, Malaysia), housed in groups of five and given 2 weeks to acclimate to the housing facility. The mice were housed in a well ventilated room maintained at a temperature of 23°C with 12 h light/dark cycles, 30%-40% humidity and had free access to standard mice chow (Specialty Feeds Pty Ltd., Glen Forrest, Australia) and tap water *ad libitum*. During housing, animals were monitored daily for health status. No adverse events were detected.

A total of 48 mice were randomly assigned into the following groups: 1) control group; 2) group that received intra-peritoneal injection of ER stress inducer, tunicamycin (Tu, 1 mg/kg, 2 injections/week for 2 weeks) and vehicle (saline, oral gavage, daily for 2 weeks); 3) group that received tunicamycin and oral administration of paeonol (20 mg/kg/day for 2 weeks) (Tu + Paeonol); 4) group that received only oral administration of paeonol (20 mg/kg/day for 2 weeks); 5) group that received tunicamycin and daily oral administration of a ROS scavenger, tempol (20 mg/kg/day) for 2 weeks (Tu + Tempol); 6) group that received tunicamycin and daily intra-peritoneal injection of ER stress inhibitor, taurine-conjugated tursodeoxycholic acid (TUDCA, 150 mg/kg/day) for 2 weeks (Tu + TUDCA). For group that do not receive tunicamycin, the same dosage of saline as tunicamycin was given 2 injections per week for two weeks via intraperitoneal injection. No animal was excluded from each experiment. The experimenters were blinded to the pharmacological treatment while processing data and making exclusion decisions. The dose of paeonol was determined from literature [25, 28, 29] and our preliminary data (S1 Fig) showed that paeonol treatment at 20 mg/kg improved endothelium-dependent relaxation in mice treated with tunicamycin.

The body weights were recorded daily during the experimental period. Systolic blood pressure (SBP) of the mice was measured at day 0, day 7 and before sacrifice (day 14) using the tail-cuff blood pressure system (NIBP Monitoring System, IITC Inc., Woodland Hills, CA, USA). The animals were restrained in a pre-warmed chamber (28–30°C) for at least 30 min before the blood pressure measurement was carried out. The arterial blood pressure measurements were performed at the same time of day (between 9 a.m. and 11 a.m.) in order to avoid the influence of the circadian cycle. The value of SBP was recorded and reported as the average of 6 successive measurements.

At the end of the treatment period, mice were anaesthetized with CO_2_ inhalation and blood samples were collected. Blood samples were centrifuged at 2500 rpm for 10 min at 4°C to obtain serum, which was immediately stored at -80°C until further use. Then, mouse aorta was isolated immediately and processed accordingly for subsequent experiments. All sections of this report adhere to the ARRIVE Guidelines for reporting animal research [30]. A completed ARRIVE guidelines checklist is included in Checklist S3.

### Functional study

The descending thoracic aorta was carefully isolated and cleaned from adjacent connective tissues and fat. The aorta was cut into rings segments, 3–5 mm long and placed in oxygenated Krebs physiological salt solution (KPSS in mM: NaCl 119, NaHCO3 25, KCl 4.7, KH2PO4 1.2, MgSO4.7H2O 1.2, glucose 11.7, and CaCl2.2H2O 2.5). Some of the rings were snap frozen in liquid nitrogen and stored in -80°C for protein analysis. Two mounting wires were threaded through the isolated mouse aorta rings and secured to two supports in a Multi Wire Myograph System (Danish Myo Technology, Aarhus, Denmark). One support was attached to a micrometer for the adjustment of vessel circumference and application of tension. The other support was attached to an isometric transducer. The fresh aortic rings were maintained at 37°C and stretched to optimal basal tension of 5 mN with continuous oxygenation of 95% O2 and 5% CO2. The rings were equilibrated for 45 min before being stimulated with 80 mM KCl to prime the tissues and then were rinsed with Krebs solution for 3 times. Once tissues were stabilised, phenylephrine (PE, 3 μM) was added to induce a sustained contraction. Endothelium-dependent relaxation (EDR) was generated by cumulative addition of acetylcholine (ACh, 3 nM to 10 μM; Sigma-Aldrich) and α2-adrenoceptor agonist, UK14304 (3 nM to 10 μM; Sigma-Aldrich). Endothelium-independent relaxation to sodium nitroprusside (SNP, 1 nM to 10 μM; Sigma-Aldrich) was also carried out. The changes of isometric tension were recorded using the PowerLab LabChart 6.0 recording system (AD Instruments, Bella Vista, NSW, Australia). Each experiment was performed on rings obtained from different mice from each group. Concentration-response curves for both endothelium-dependent and -independent relaxation were expressed as the percentage of reduction in contraction induced by PE before the application of ACh, UK14304 or SNP independently. The maximum effect (R_max_) and the concentration inducing 50% of R_max_ (pEC_50_) were determined from the cumulative concentration-response curves.

### Cell culture

Human umbilical vein endothelial cells (HUVECs, Lonza, Basel, Switzerland, No. CC-2517) were grown in endothelial cell growth medium (EGM, Gibco, Invitrogen) supplemented with 10% FBS, 100 U/mL penicillin, 100 μg/mL streptomycin and endothelial cell growth supplement (50 μg/L, BD Transduction Laboratory, San Diego, CA, USA). The cells were cultured in a humidified atmosphere containing 5% CO2 at 37°C. Cells from passages between 4 and 6 were used. Once the cells reached 90% confluency, experiments were performed. The cells were cultured to 7 experimental groups (n = 4–5) for 16 hours. The experimental groups are: 1) control, 2) tunicamycin, (ER stress inducer, 0.5 μg/mL), 3) tunicamycin + paeonol (0.1 μM), 4) paeonol (0.1 μM), 5) tunicamycin + TUDCA (ER stress inhibitor, 10 μM), 6) Tunicamycin + tempol (ROS scavenger, 100 μM), 7) tunicamycin + TUDCA + tempol. The concentration and time duration were chosen based previously reported effective concentration and time [27, 31].

### Detection of ROS formation in *en face* endothelium of mouse aortas and HUVECs

The level of oxidative stress in the *en face* endothelium of mouse aorta and HUVECs was assessed using dihydroethidium (DHE) dye by confocal microscopy [27]. The treated HUVECs and aortic rings were incubated with DHE (5 μM, Invitrogen, Carlsbad, CA, USA) for 15 min at 37°C in normal physiological saline solution (NPSS, composition in mM: NaCl 140, KCl 5, CaCl_2_ 1, MgCl_2_ 1, glucose 10 and HEPES 5) with pH 7.4. After incubation, cells and the aortic rings were rinsed 3 times with NPSS. The aorta rings were cut open, and the endothelium was placed upside down between two coverslips on the microscope. Fluorescence intensity was captured by confocal microscope Leica TCS SP5 II (Leica Microsystems, Mannheim, Germany) with 515-nm excitation and 585-nm long pass filter. Background autofluorescence of elastin in aortic rings were taken at excitation 488 nm and emission 520 nm separately to avoid overlapping of the emission spectrum. DHE fluorescence intensity was analyzed by Leica LAS-AF software version 2.6.0.7266 as represented by the fold change in fluorescence intensity relative to the control group.

### Detection of vascular superoxide formation

Lucigenin-enhanced chemiluminescence method was used to quantify the vascular superoxide anion production as previously described [27]. Briefly, aortic rings from each groups was pre-incubated for 45 min at 37°C in Krebs-HEPES buffer (in mM: NaCl 99.0, NaHCO3 25, KCl 4.7, KH2PO4 1.0, MgSO4 1.2, glucose 11.0, CaCl22.5 and Na-HEPES 20.0) in the presence of diethylthiocarbamic acid (DETCA, 1 mM) and β-nicotinamide adenine dinucleotide phosphate (β-NADPH, 0.1 mM). DECTA was used to inactivate superoxide dismutase (SOD) while β-NADPH was used as a substrate for NADPH oxidase. Inhibitor of NADPH oxidase, diphenylene iodonium (DPI; 5 mM) was added for the positive control. Before measurement, a 96-well Optiplate containing lucigenin (5 mM) and β-NADPH (0.1 mM) in 300 ml of Krebs-HEPES buffer per well was loaded into the Hidex plate CHAMELEONTM V (Finland). Background photo emission was measured with 30 seconds intervals over 20 min. The rings were then transferred into wells and measurement was taken again. Upon completion of measurement, the rings were dried for 48 h at 65°C and weighed. The data are expressed as average counts per mg of vessel dry weight.

### Western blotting

Treated mouse aortas or HUVECs were homogenized in ice-cold RIPA lysis buffer containing leupeptin 1 μg/mL, aprotinin 5 μg/mL, PMSF 100 μg/mL, sodium orthovanadate 1 mM, EGTA 1 mM, EDTA 1 mM, NaF 1 mM, and β-glycerolphosphate 2 mg/mL. The lysates were centrifuged at 20,000 g for 20 min at 4°C. The supernatant was collected and the protein concentrations were measured using Lowry assay (Bio-Rad Laboratories, Hercules, CA, USA). Protein samples (15 μg) were separated with 10% SDS-polyacrylamide gels and transferred to an immobilon-P polyvinylidene difluoride membrane (Millipore, Billerica, MA, USA) using wet transfer (Bio-Rad) at 4°C. Non-specific binding sites were blocked by 3% BSA in 0.05% Tween-20 phosphate-buffered saline, and then incubated at 4°C overnight with primary antibodies against GRP78 (1:1000, Santa Cruz), activating transcription factor 6 (ATF6; 1;1000, Abcam, Cambridge, UK), phosphorylated eukaryotic initiation factor 2 alpha (eIF2α) at Ser^52^ (1;1000, Cell Signalling), eIF2α (1;1000, Cell Signalling), phosphorylated endothelial nitric oxide synthase (eNOS) at Ser^1176^ (1;1000, Abcam), eNOS (1;1000, BD Transduction laboratory, San Diego, CA, USA), Nox 2 (1;1000, Abcam, Cambridge, UK), nitrotyrosine (1;1000, Abcam, Cambridge, UK) and GAPDH (Santa Cruz). The membrane were washed with TBS-T, followed by incubation with appropriate horseradish peroxidase-conjugated secondary antibodies (DakoCytomation, Carpinteria, CA, USA) for 2 h at room temperature. The membranes were developed with an enhanced chemiluminescence detection system (ECL reagents, Millipore Corporation, Billerica, MA), and exposed to X-ray films. Densitometric analysis was performed using Quantity One analysis software (Bio-Rad). Equal protein loading was verified with use of GAPDH as housekeeping protein. The respective protein expression levels for GRP78, ATF6, NOX2 and nitrotyrosine were normalized to GAPDH, peNOS to eNOS, peIF2α to eIF2α, and then compared with control.

### Measurement of vascular nitrate/nitrite level

The total nitrate/nitrite level was detected in the aorta using a Nitrate/Nitrite Colorimetric Assay Kit (Cayman Chemical Company, Ann Arbor, MI, USA) according to the manufacturer’s protocol. The absorbance was measured using Hidex plate CHAMELEONTM V (Turku, Finland) and compared with a standard nitrite curve at 540 nm. The results are expressed in μM.

### Data analysis

Results are represented as means ± SEM from n experiments. Concentration-response curves were fixed to a sigmoidal curve using non-linear regression using statistical software GraphPad Prism version 4 (GraphPad Software Inc., San Diego, CA, USA). Statistical significance was determined using two-tailed Student's t-test for comparison of two group and one-way ANOVA followed by Bonferroni multiple comparison tests when more than two treatments were compared. Results with P values <0.05 were considered statistically significant.

## Results

### General parameters; body weight and systolic blood pressure

Mice treated with tunicamycin showed a significant increase in systolic blood pressure compared with the control group (125.20±3.01 versus 94.03±3.36 mmHg; P<0.05) at the end of two weeks. This increase was significantly reduced by co-treatment with paeonol (103.70±6.83 mmHg), ER stress inhibitor, TUDCA (103.70±6.19 mmHg) and tempol (98.84±1.53 mmHg) as shown in Fig 1B. Mice treated with tunicamycin for two weeks demonstrated a reduction in body weight, and it was improved following co-treatment with paeonol, TUDCA and tempol (Fig 1C). There were no significant changes in both body weight and systolic blood pressure between the paeonol only and control group (Fig 1B & 1C).

### Paeonol improved tunicamycin-induced endothelial dysfunction in mouse aorta

**Fig 2:**
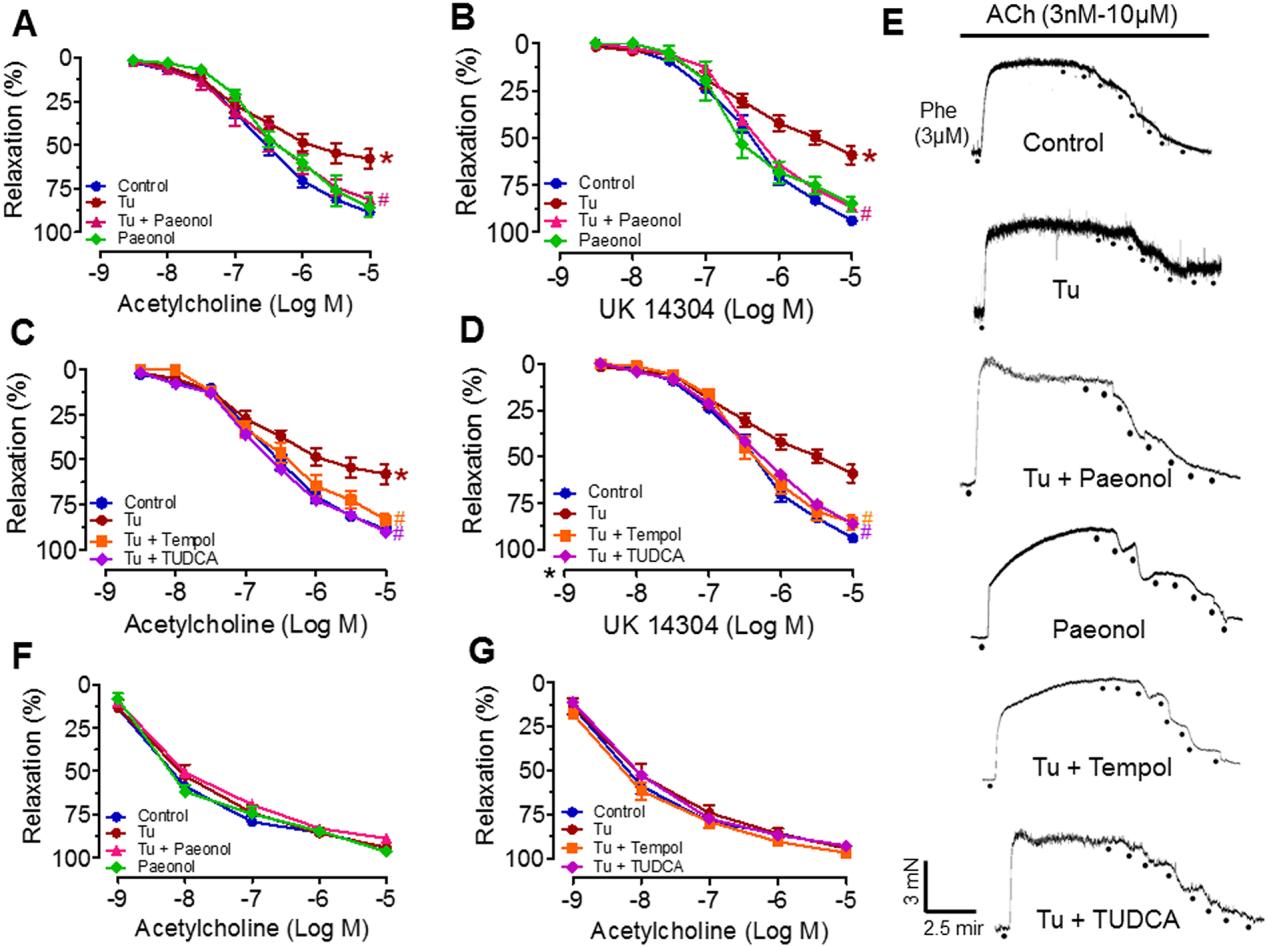
Endothelium-dependent relaxations induced by (A&C) acetylcholine (ACh) or (B&D) UK14304, (E) its representative traces and (F&G) endothelium-independent relaxations induced by sodium nitroprusside (SNP) of aortae rings in mice with or without 2 weeks chronic treatment of tunicamycin (Tu, 1 mg/kg, 2 injections/week/i.p.), paeonol (20 mg/kg/day/oral gavage), tempol (20 mg/kg/day/oral gavage) and TUDCA (150 mg/kg/day/i.p.). Results are means ± SEM of 6–7 experiments. * P < 0.05 when compared with control, #p<0.05 when compared with tunicamycin.

To determine the role of paeonol treatment in ER stress-induced endothelial dysfunction in mice, we examined EDR and endothelium-independent relaxation produced by ACh, UK14304 and SNP in aorta respectively in a concentration dependent manner. Mice treated with tunicamycin for 2 weeks displayed attenuated EDR (ACh and UK14304) compared to the aorta from the control group. Chronic treatment with either paeonol or TUDCA significantly improved EDR impaired by tunicamycin (Fig 2A–2E, Table 1). The role of vascular oxidative stress in mice induced by tunicamycin was evaluated following chronic treatment with tempol, a superoxide scavenger. Co-treatment with tempol prevented the tunicamycin-induced impairment of relaxations to ACh in mice (Fig 2C–2E, Table 1). However, the EDR of the paeonol only group were similar to those of the control group (Fig 2A & 2B). Sodium nitroprusside-induced endothelium-independent relaxation was similar in all treatment groups, suggesting the sensitivity of vascular smooth muscle to NO remained intact (Fig 2F and 2G, Table 1).

**Table 1:** Agonist sensitivity (pEC_50_) and % maximum response (R_max_) of endothelium-dependent vasodilators, acetylcholine (ACh), UK14304 and endothelium-independent vasodilator sodium nitroprusside (SNP), in isolated aorta from C57BL/6J mice treated with tunicamycin (Tu), paeonol, tempol and TUDCA for 2 weeks. Results are means ± SEM (n = 6–7).

Groups	ACh		UK14304		SNP	
	pEC_50_ (log M)	R_max_ (%)	pEC_50_ (log M)	R_max_ (%)	pEC_50_ (log M)	R_max_ (%)
Control	6.64 ± 0.07	89.73 ± 1.64	6.42 ± 0.07	93.30 ± 1.95	7.22 ± 0.52	94.30 ± 1.66
Tu	6.87 ± 0.18	55.20 ± 4.29*	6.44 ± 0.19	60.50 ± 5.40*	7.30 ± 0.26	94.12 ± 1.93
Tu +Paeonol	6.72 ± 0.18	85.08 ± 1.99#	6.40 ± 0.12	86.67 ± 2.95#	7.24 ± 0.52	95.91 ± 2.97
Paeonol	6.47 ± 0.12	90.10 ± 2.16	6.67 ± 0.10	91.24 ± 2.05	7.25 ± 0.43	92.76 ± 2.45
Tu + Tempol	6.72 ± 0.14	84.50 ± 3.49#	6.50 ± 0.07	87.83 ± 4.22#	7.39 ± 0.28	96.82 ±0.94
Tu + TUDCA	6.74 ± 0.15	85.75 ± 4.69#	6.36 ± 0.14	85.05 ± 3.92#	7.40 ± 0.18	96.51 ± 2.22

* P<0.05 compared with control,

^#^ P<0.05 vs. tunicamycin.

### Paeonol inhibited ER stress-induced oxidative stress in mouse aorta

**Fig 3:**
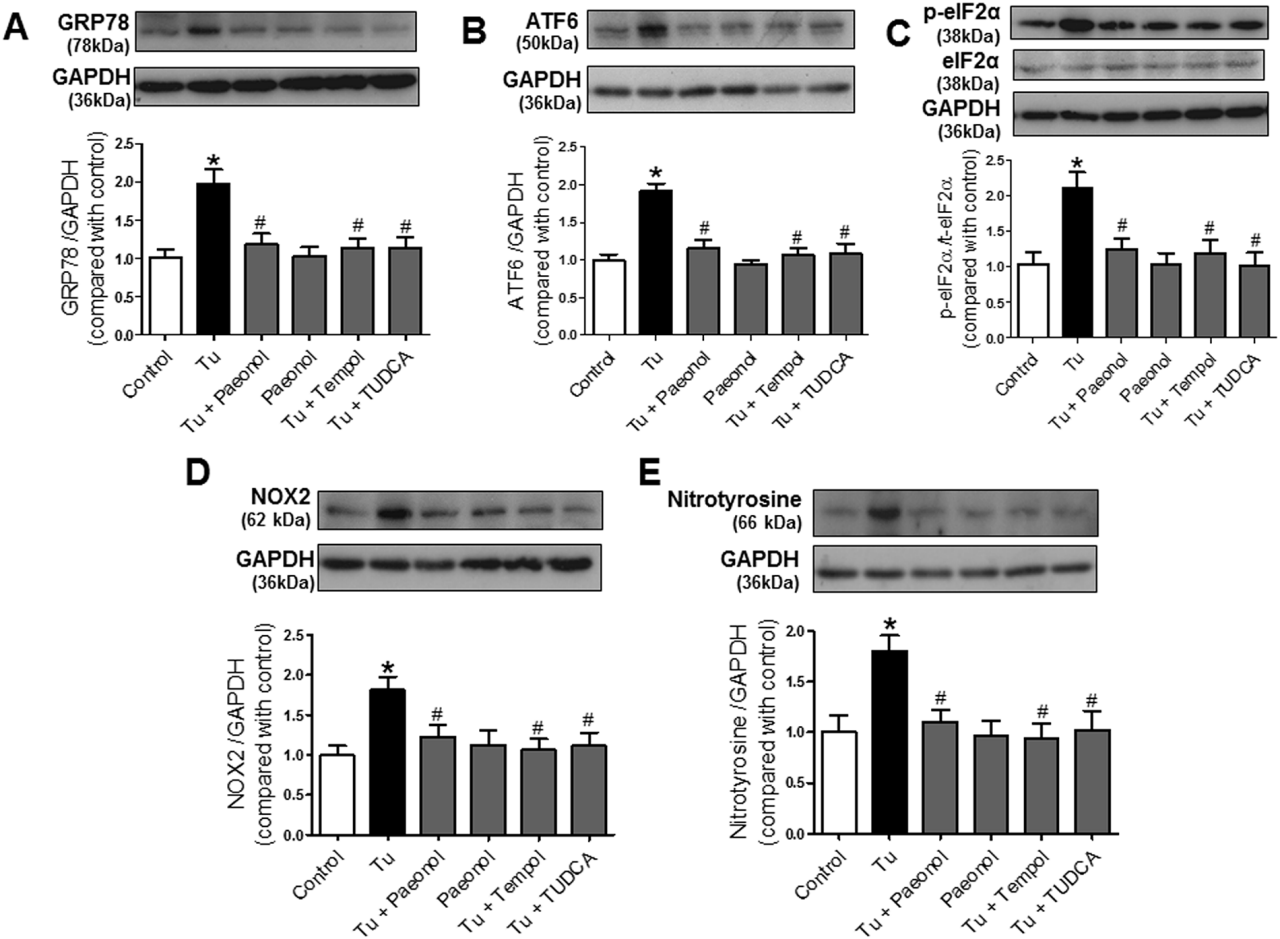
Western blot and quantitative data showing the ER stress markers, (A) glucose-regulated protein 78 (GRP78), (B) activating transcription factor-6 (ATF6), (C) phosphorylation of eukaryotic translation initiation factor 2 alpha (eIF2α) and oxidative stress markers, (D) NOX2 and (E) nitrotyrosine in C57BL/6J mice treated 2 weeks with or without tunicamycin (Tu, 1 mg/kg, 2 injections/week/i.p.), paeonol (20 mg/kg/day/oral gavage), tempol (20 mg/kg/day/oral gavage) and TUDCA (150 mg/kg/day/i.p.). Results are means ± SEM of 6–7 separate experiments. *P<0.05 compared with control, # P<0.05 compared with tunicamycin.

We next explored the effect of chronic treatment with paeonol on ER stress-associated proteins. Glucose-regulated protein 78 (GRP78) (Fig 3A), activating transcription factor-6 (ATF6) (Fig 3B) and phosphorylation of eukaryotic translation initiation factor 2 alpha (eIF2α) (Fig 3C) proteins were all elevated in mice treated by tunicamycin, and were reversed following co-treatment with paeonol and TUDCA. Additionally, co-treatment with either paeonol or tempol inhibited the tunicamycin-stimulated up-regulation of NADPH subunits, NOX2 and nitrotyrosine (marker for peroxynitrate, an index for increased oxidative stress) in mice compared with the control group (Fig 3D and 3E). No significant changes were observed between the control and the paeonol only groups (Fig 3A–3E).

### Paeonol reduced the superoxide production in mouse aorta

**Fig 4:**
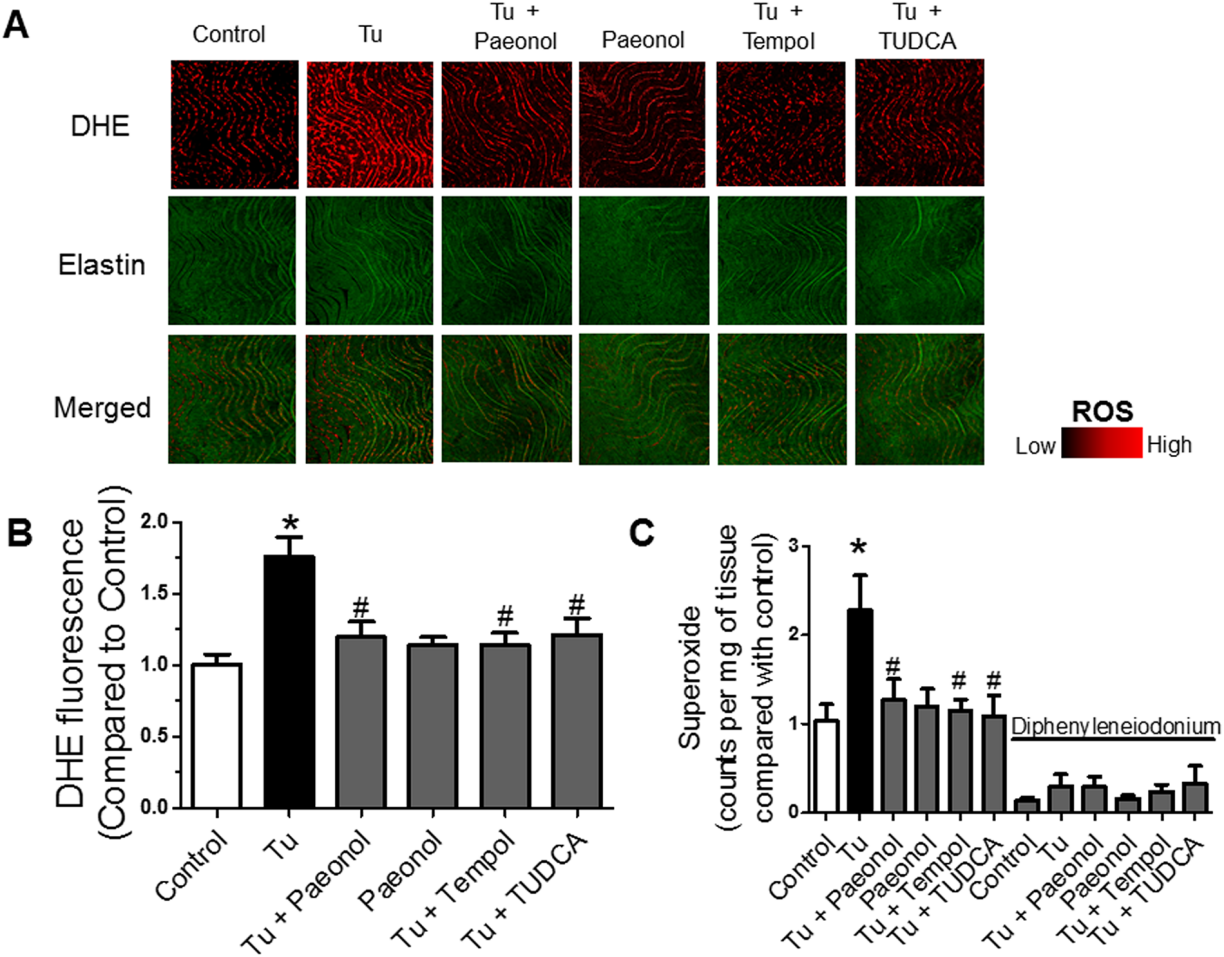
Representative images and summarized results of superoxide production measured by (A&B) DHE fluorescence in the *en face* endothelium of aorta and (C) lucigenin-enhanced chemiluminescence method in the aorta of C57BL/6J mice of all groups. Red: DHE fluorescence (excitation: 515 nm) in the nucleus. Green: autofluorescence of elastin underneath the endothelium (excitation: 488 nm). Lower panel, merged. Bar: 100μm. The NADPH oxidase inhibitor diphenyleneiodonium (DPI, 10 mM) abolished the generation of superoxide anion. Results are means + SEM of 6–7 separate experiments. *P<0.05 compared with control, # P<0.05 compared with tunicamycin.

Next, we determined ROS level in mouse aorta arteries. ROS formation in *en face* endothelium and O_2_^−^ level was markedly increased mice treated for 2 weeks with tunicamycin compared to control group as reflected by the intensity of DHE fluorescence staining (Fig 4A and 4B) and lucigenin-enhanced chemiluminescence (LEC) (Fig 4C) respectively. Co-treatment with paeonol or TUDCA reduced the tunicamycin-stimulated ROS. Similarly, chronic treatment with ROS scavenger, tempol normalized the elevated ROS production in mice treated with tunicamycin.

In accordance to the DHE staining on the *en face* of endothelium in mouse aorta, HUVECs treated with tunicamycin showed an increased in ROS production (S2A Fig) and NOX2 protein up-regulation (S2B Fig), which were reduced with co-incubation with paeonol. Similarly, co-incubation with tempol, TUDCA, tempol + TUDCA respectively reversed the adverse effect of tunicamycin. The ROS level in paeonol only group were similar to the control group in both HUVECs (S2A–S2C Fig) and mouse aorta (Fig 4A–4C).

### Paeonol enhanced nitric oxide bioavailability in mouse aorta

**Fig 5:**
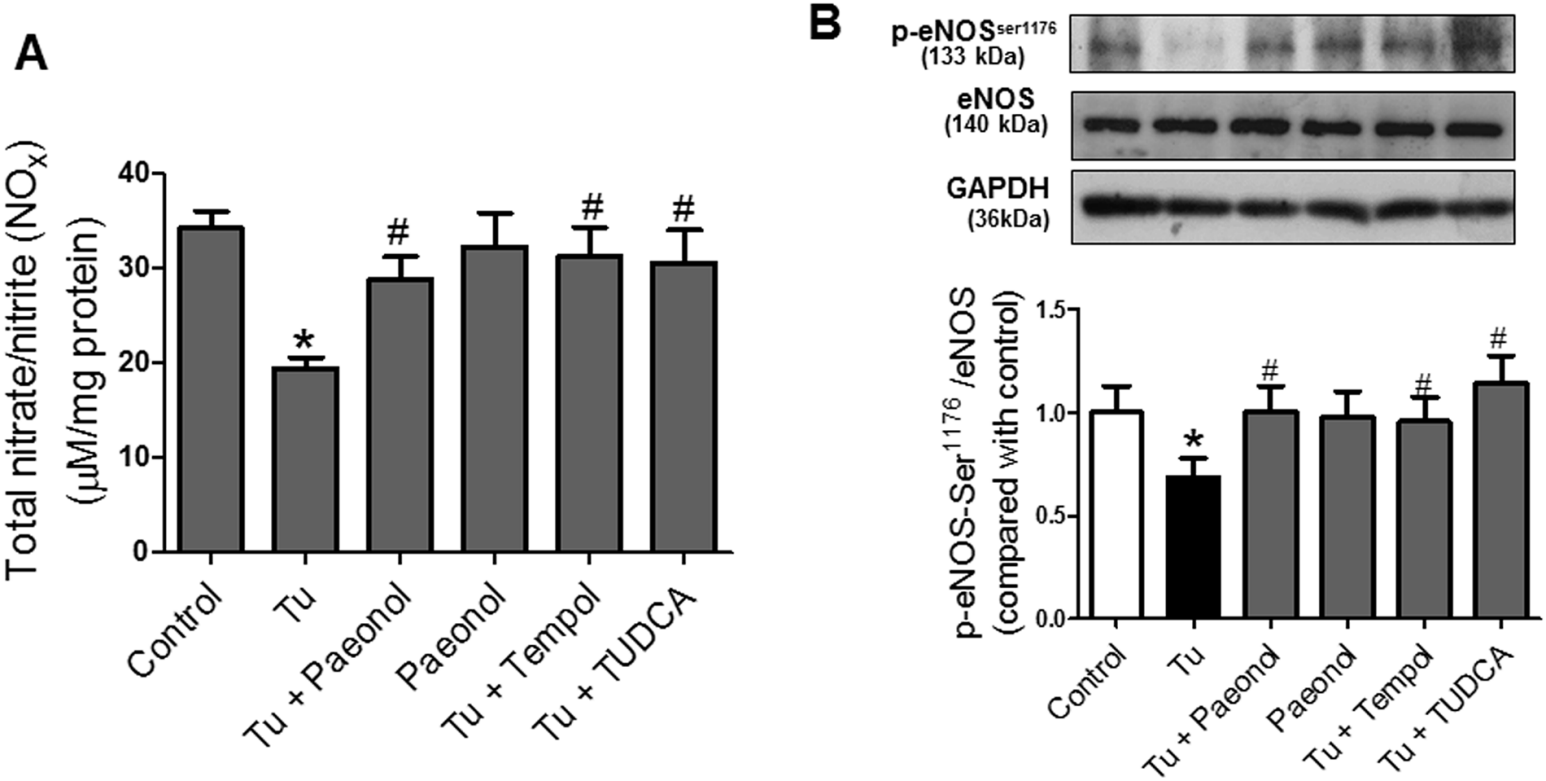
Paeonol treatment (20 mg/kg/day/oral), tempol (20 mg/kg/day/oral) and TUDCA (150 mg/kg/day/i.p.) for two weeks increased (A) tissue total nitrite/nitrate level and (B) phosphorylation of eNOS which was reduced in C57BL/6J mice induced with intra-peritoneal injection of tunicamycin (Tu, 1 mg/kg, 2 injections/week for two weeks) as measured by colorimetric assay kit and western blot respectively. Data are expressed as means ± SEM (n = 6–7). *p ≤ 0.05 compared to control; #p ≤ 0.05 compared to mice induced with tunicamycin.

Tunicamycin treated mice displayed significant decrease in tissue total nitrate/nitrite level compared to the control mice (Fig 5A). The effect of tunicamycin was reversed by chronic co-treatment with paeonol, TUDCA and tempol respectively. In addition, chronic paeonol treatment promoted phosphorylation of eNOS at Ser^1176^ in aortas which was reduced in tunicamycin treated mice. Chronic tempol and TUDCA treatment similarly increased phosphorylation of eNOS at Ser^1176^ compared to mice induced with tunicamycin (Fig 5B). There were no significant differences in mice treated with paeonol only and control group (Fig 1B & 1C).

## Discussion

The present study demonstrates that chronic treatment with paeonol *in vivo* confers vascular protection by alleviating ER stress and oxidative stress. We observed increased systolic blood pressure, reduction of body weight, impairment of endothelium-dependent relaxation, up-regulations of ER stress markers, increased ROS generation and reduced nitric oxide (NO) bioavailability in aortae following treatment with tunicamycin in C57BL/6J mice. These were reversed by chronic co-administration with paeonol, TUDCA or tempol, respectively.

Prolonged ER stress will lead to advanced lesional macrophage death, plaque necrosis and increases vascular smooth muscle contractility resulting in increased blood pressure [6, 10]. Our results revealed that mice treated with tunicamycin showed elevated blood pressure and reduction in body weight, which is in agreement with previously reported literature [10, 32]. These elevated blood pressure and reduction in body weight were normalised by treatment of paeonol chronically for two weeks. Previous study reported that ER stress may increase blood pressure by increasing cardiac output and peripheral vascular resistance [10]. In addition, ER stress has been reported in hypertensive patients [33], animals with metabolic syndrome [34], high salt intake-induced hypertensive rats [35] and angiotensin II-induced hypertensive mice [36]. In fact, recent *in vivo* findings have shown that ER stress inhibitor, TUDCA reduced blood pressure and improved vascular activity in spontaneously hypertensive rats (SHRs) through inhibition of ER stress [37]. Furthermore, hypertension in human is associated with decreased NO bioavailability and an increased in oxidative stress [20]. It has been shown that oxidative stress is the key contributor in the pathogenesis of hypertension [21, 38]. Oxidative stress can impact vascular tone leading to endothelial dysfunction [39, 40]. ROS promotes vascular cell proliferation and migration, inflammation and apoptosis, as well as extracellular matrix alterations [41, 42]. Inhibition of ER stress in hypertension improved macrovascular endothelial function by transforming growth factor-β1 (TGF-β1)-dependent mechanism and microvascular endothelial function by an oxidative stress-dependent mechanism [36]. The anti-hypertensive effects of paeonol are comparable to those produced by ER stress inhibitor (TUDCA) and antioxidant (tempol) or both, suggesting that it may work by inhibiting ER stress-mediated oxidative stress pathway [43, 44]. These results are in agreement with Al-Magableh and co-workers (2015) findings which showed that hydrogen sulfide reduced blood pressure in angiontensin II-induced hypertensive mice through inhibition of vascular oxidative stress [45].

ER stress has a negative impact on vascular function as treatment with tunicamycin for several weeks reduced ACh-induced endothelium-dependent relaxation in large and small arteries [36, 46]. ER stress also triggers inflammatory signalling mechanism [47] and reduced phosphorylation of eNOS causing endothelial dysfunction [48]. ER stress through the activation of NFkβ and transforming growth factor beta 1 (TGFβ-1), contribute to an increase in ROS generation, which also culminates in vascular dysfunction and development of hypertension [49]. Tunicamycin caused less impairment of the EDR to ACh in aorta of *p47phox−/−* mice than wild type control mice suggesting association of ER stress to enhanced NADPH oxidase-ROS activity [48]. In a stressed ER, dysregulated disulfide bond formation and breakage may result in ROS accumulation and induced oxidative stress [50]. In addition, some UPR components such as CHOP may promote apoptosis, released ROS and impaired the endothelium [51]. In agreement with our earlier findings [27], *in vivo* treatment with paeonol, TUDCA and tempol reversed the impaired endothelium-dependent relaxations to ACh and UK14304, an α_2_ adrenoceptor agonist in aorta isolated from tunicamycin treated mice. Paeonol is known for its anti-inflammatory effects [52, 53]. The attenuation of ER stress-induced inflammation may also have contributed to the protective effects of paeonol against ER stress-related injuries [54, 55]. Previous studies have shown that paeonol induces vasodilatation of the rat mesenteric arteries by inhibiting extracellular Ca^2+^ influx and intracellular Ca^2+^ release [56]. Additionally, our *in vivo* results revealed that mice treated with tunicamycin showed upregulation of ER stress proteins (phosphorylation of eIF2α, ATF6 and GRP78) which were reversed by treatment with paeonol, TUDCA and tempol. Taken together, the results indicate that paeonol improves EDRs probably through inhibition of ER stress.

Oxidative stress and increased ROS production is an integral component of acute and chronic states of UPR signalling [13]. Increased unfolded proteins in the ER stimulates Ca^2+^ leakage into the cytosol and further augment oxidative phosphorylation of the electron transport chain, increase cytochrome c release impairing electron transfer, altering mitochondrial membrane potential and increasing the generation of ROS [57, 58]. Recent evidence reveals that under ER stress, ROS production is increased via enzymes of the NADPH oxidase (NOX) family, especially via NOX2, which is involved in blood pressure regulation [59] and augmentation of proapoptotic signalling [60]. Cholesterol or 7-ketocholesterol-induced ER stress promote pathophysiology of various cardiovascular diseases, including heart failure and oxidative shift in macrophages, which are suppressed by NOX2 siRNA [7]. This eventually decreases the bioavailability of NO and ultimately leads to endothelial dysfunctions [61]. ROS production is also increased by electron leakage from ER stress-associated p450 2E1 activation, a pro-oxidant protein [62]. In agreement to these previous studies, treatment with paeonol inhibited the tunicamycin-induced vascular expression of NOX2 and nitrotyrosine, a marker for peroxynitrite in a similar manner to tempol, a free radical scavenger. The alleviation of ROS following paeonol treatment was accompanied with increased bioavailability of NO. Paeonol treatment prevent*s* ER stress induced by tunicamycin in the mice by reducing ROS production via inhibition of NOX2 and nitrotyrosine. Similarly, paeonol improved EDRs measured in the isolated mouse aorta to a similar extend as following treatment with tempol and TUDCA. Paeonol likely work on the ROS signalling, extracellular signal-regulated kinases (ERK), Ca^2+^ mechanisms such as ischemia-reperfusion (I/R) preconditioning pathways to protect ER from oxidative stress [63, 64]. Additionally, paeonol has been was reported to enhance antioxidant defence via nuclear factor erythroid 2-related factor 2 (Nrf2) activation *in vivo* [65].

**Fig 6:**
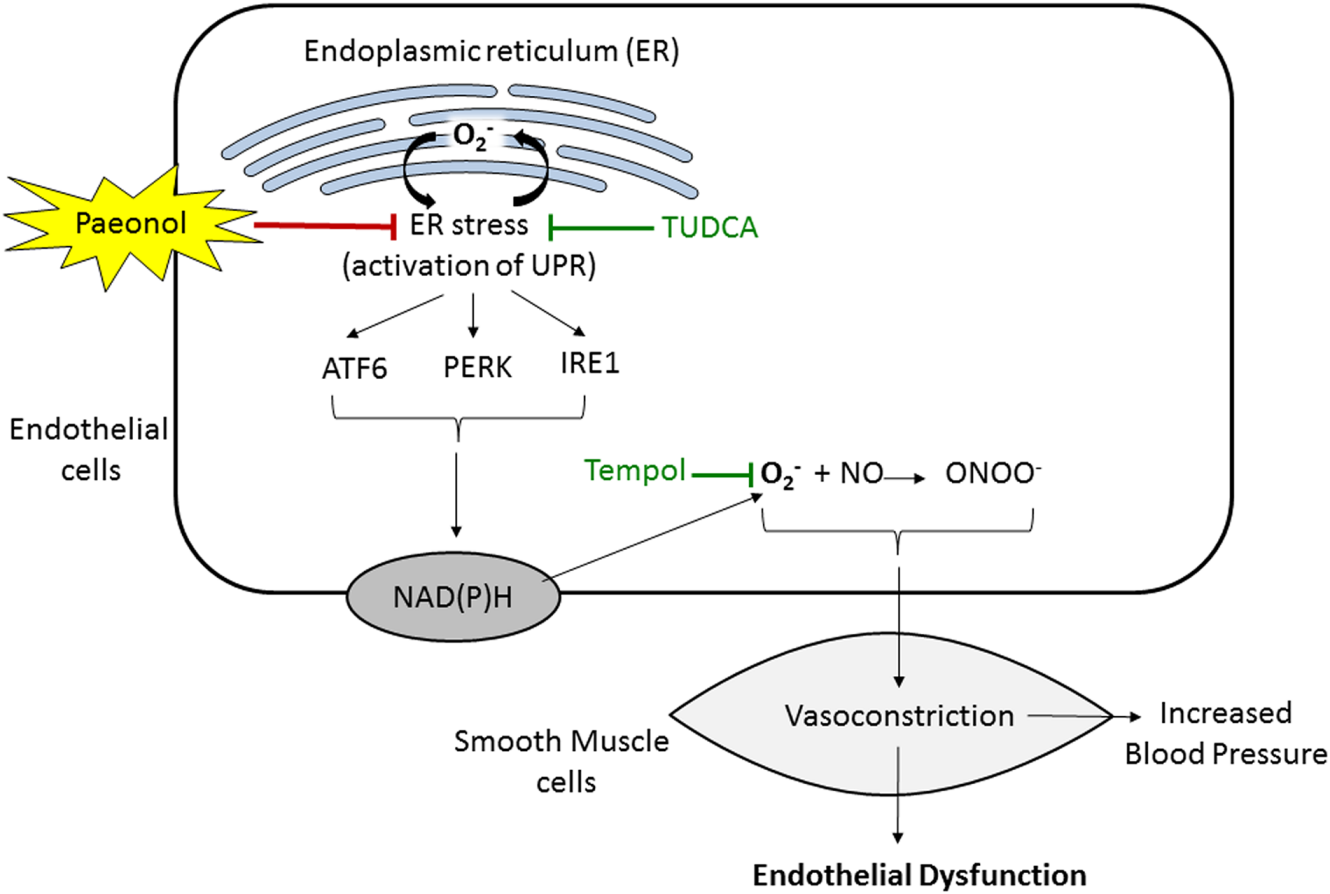
Schematic diagram showing chronic paeonol treatment alleviates endoplasmic reticulum (ER) stress, inhibits reactive oxygen species (ROS) production and improves NO bioavailability. Subsequently, it reverses the endothelial dysfunction and normalised blood pressure in mice. ER, endoplasmic reticulum; ATF6, activating transcription factor 6; PERK, PKR-like eukaryotic initiation factor 2 kinase; IRE1, inositol-requiring enzyme-1; O^2-^, superoxide; NO, nitric oxide; ONOO^−^, peroxynitrite; NOS; NAD(P)H, Nicotinamide adenine dinucleotide phosphate.

In summary, the present results demonstrate that chronic administration of paeonol in tunicamycin-induced ER stress in mice confers protection against endothelial dysfunction and normalised blood pressure by alleviating ER stress-induced oxidative stress (Fig 6). The present data provides further evidence supporting the potential use of paeonol as novel therapeutic agents or health supplements for patients with ER-stress related cardiovascular diseases, particularly in the treatment of hypertension.

## Supporting information

S1 Checklist ARRIVE guidelines checklist. (PDF)

S1 Fig Paeonol improved endothelium dependant relaxation of mice aorta in a dose dependant manner. Endothelium-dependent relaxations induced by acetylcholine of aortae rings in mice with or without 2 weeks chronic treatment of tunicamycin (Tu, 1 mg/kg, 2 injections/week/i.p.), paeonol (10 mg/kg/day/oral gavage) or paeonol (20 mg/kg/day/oral gavage). Results are means ± SEM of 6 experiments. *P < 0.05 when compared with control, #p<0.05 when compared with tunicamycin.
(TIF)

S2 Fig Paeonol reduced superoxide production induced by tunicamycin in HUVECs cells. (A) Representative images and (B) summarized results of superoxide production measured by DHE in HUVECs incubated with tunicamycin (Tu, 0.5 μg/ml) for 16 hours. Tunicamycin increased superoxide production but its effect was reduced by co-incubation with paeonol (0.1 μM), tempol (ROS scavenger, 100 μM), TUDCA (ER stress inhibitor, 10 μM) and both tempol + TUDCA. Bar: 100μm. Results are means ± SEM of 4 separate experiments. *P<0.05 compared with control, # P<0.05 compared with tunicamycin.
(TIF)
