# Supplementary figures and images for: Chronic treatment with paeonol improves endothelial function in mice through inhibition of endoplasmic reticulum stress-mediated oxidative stress
Source: PLoS One. 2017 May 31;12(5):e0178365. doi: 10.1371/journal.pone.0178365 (PMC5451063; doi:10.1371/journal.pone.0178365)

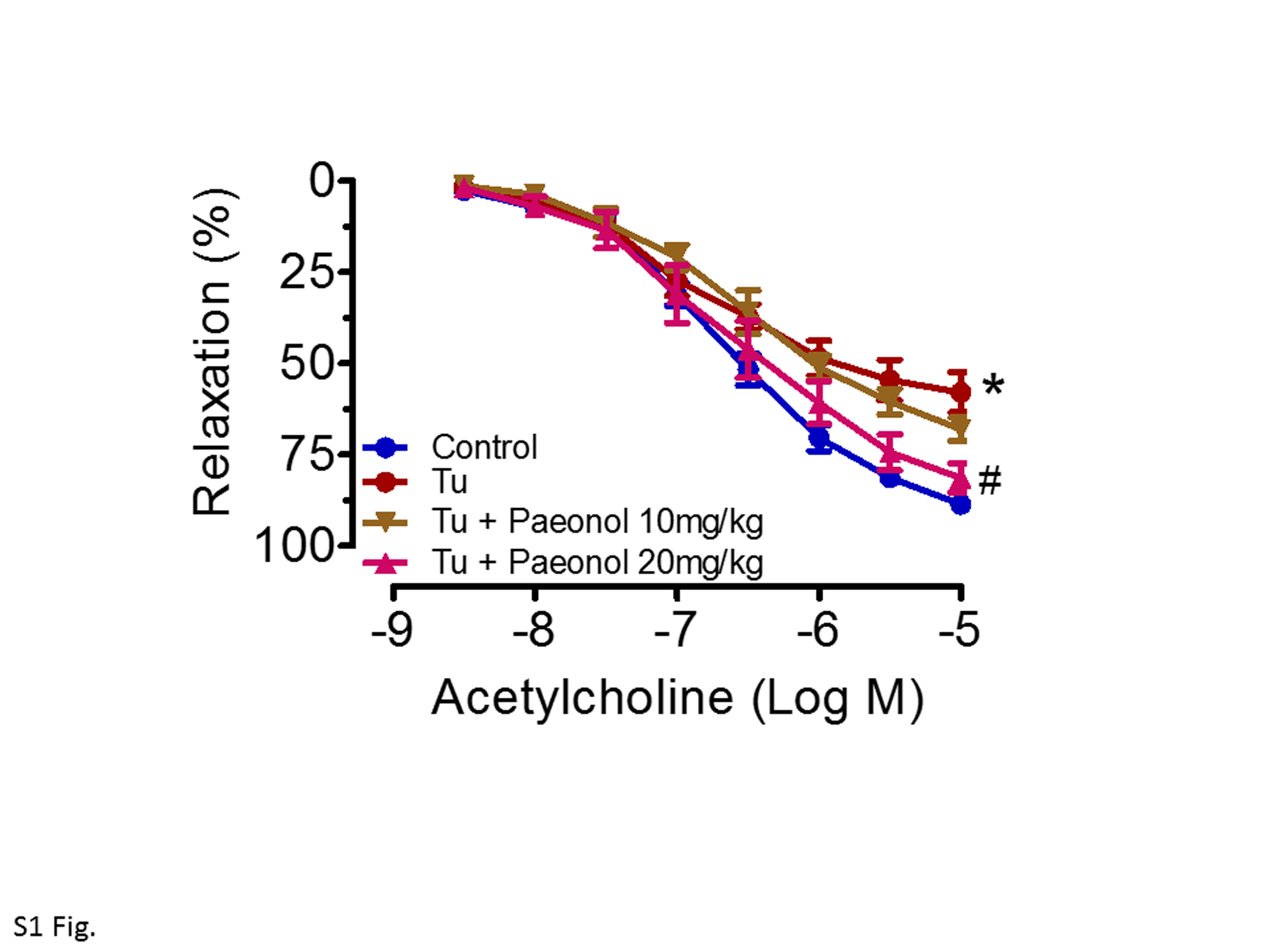

Supplement: S1 Fig — Endothelium-dependent relaxations induced by acetylcholine of aortae rings in mice with or without 2 weeks chronic treatment of tunicamycin (Tu, 1 mg/kg, 2 injections/week/i.p.), paeonol (10 mg/kg/day/oral gavage) or paeonol (20 mg/kg/day/oral gavage). Results are means ± SEM of 6 experiments. *P < 0.05 when compared with control, #p<0.05 when compared with tunicamycin. (TIF) [file pone.0178365.s002.tif]

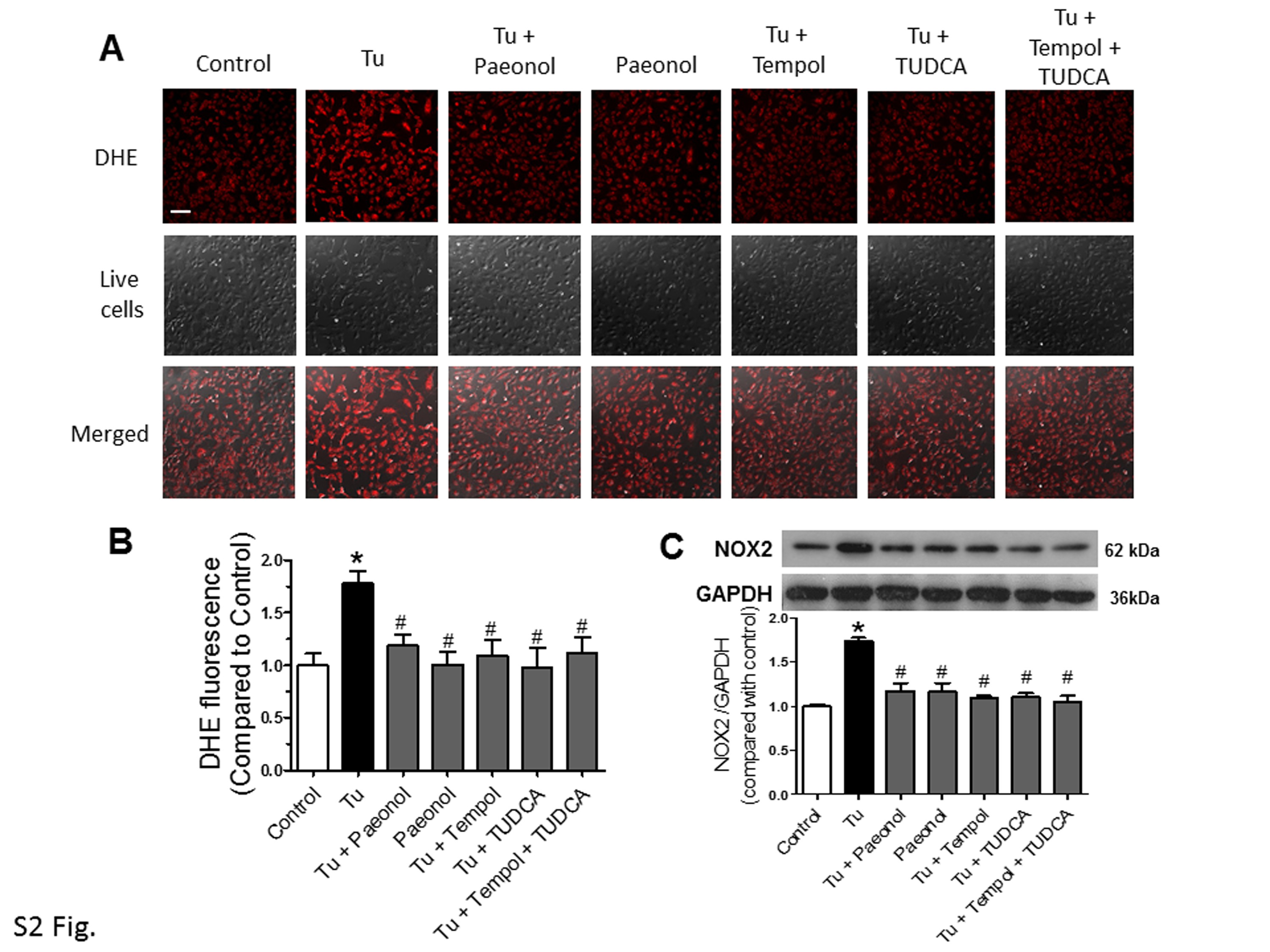

Supplement: S2 Fig — (A) Representative images and (B) summarized results of superoxide production measured by DHE in HUVECs incubated with tunicamycin (Tu, 0.5 μg/ml) for 16 hours. Tunicamycin increased superoxide production but its effect was reduced by co-incubation with paeonol (0.1 μM), tempol (ROS scavenger, 100 μM), TUDCA (ER stress inhibitor, 10 μM) and both tempol + TUDCA. Bar: 100μm. Results are means ± SEM of 4 separate experiments. *P<0.05 compared with control, # P<0.05 compared with tunicamycin. (TIF) [file pone.0178365.s003.tif]
